# 1L NER1006 can improve rates of adequate and high-quality bowel cleansing in the right colon: a post hoc analysis of two randomised clinical trials

**DOI:** 10.1186/s12876-022-02106-2

**Published:** 2022-01-25

**Authors:** Jonathan Manning, Juha Halonen, Sandra Jose Cheriyamkunnel, Marco Antonio Álvarez-González

**Affiliations:** 1 Borders General Hospital, NHS Borders, Melrose, Berwickshire, UK; 2 Norgine Ltd, Medical Affairs, Widewater Place Moorhall Road, Harefield, Uxbridge, UB9 6NS UK; 3 Hospital del Mar, Digestive Diseases, Barcelona, Spain

**Keywords:** Bowel preparation, Bowel cleansing, Colonoscopy, Colorectal cancer, Low volume, Polyethylene glycol, Right colon

## Abstract

**Background:**

The right colon is difficult to cleanse compared with other colon segments. This post hoc analysis of two randomised clinical trials (MORA and NOCT) examined whether 1L polyethylene glycol (PEG) NER1006 and two mid-volume alternatives could improve adequate and high-quality cleansing in the right colon among patients with complete cleansing assessments.

**Methods:**

Patients received NER1006 (N2D), 2L PEG plus ascorbate (2LPEG) or oral sulphate solution (OSS) as a 2-day evening/morning split-dosing regimen or NER1006 as a same-day morning-only dosing regimen (N1D). Patients had full segmental scoring assigned by treatment-blinded central readers using the Harefield Cleansing Scale. The right colon adequate (score ≥ 2) and high-quality (score ≥ 3) cleansing success of NER1006 (N2D and N1D) versus 2LPEG and OSS was analysed individually and as pooled groups (N2D vs. 2LPEG/OSS). We assessed the comparative right colon cleansing rates of the N2D versus 2LPEG/OSS in overweight males. We also performed a multivariable regression analysis to examine factors affecting cleansing in the right colon.

**Results:**

A total of 1307 patients were included. Pooled N2D showed significantly improved rates of adequate-level cleansing in the right colon compared with 2LPEG (97.5% [504/517] vs. 94.6% [246/260]; *p* = 0.020) and OSS (97.5% [504/517] vs. 93.8% [244/260]; *p* = 0.006). In MORA, the rate of adequate right colon cleansing did not significantly differ between N1D and 2LPEG (95.2% [257/270] vs. 94.6% [246/260]; *p* = 0.383). The rate of right colon high-quality cleansing was significantly improved with N2D or N1D vs. 2LPEG (*p* < 0.001 for both), and was numerically higher with N2D versus OSS (*p* = 0.11). In overweight males, NER1006 delivered numerically higher adequate (*p* = 0.398) and superior high-quality (*p* = 0.024) cleansing rates versus 2LPEG/OSS. Multivariable regression analysis showed NER1006 was associated with adequate and high-quality cleansing (*p* = 0.031 and *p* < 0.001), while time between preparation and colonoscopy was negatively associated (*p* = 0.034 and *p* = 0.006).

**Conclusions:**

NER1006 delivered improved rates of adequate and high-quality right colon cleansing compared with 2LPEG and OSS. The increased rate of high-quality cleansing with NER1006 versus its comparators was also seen in overweight males.

**Supplementary Information:**

The online version contains supplementary material available at 10.1186/s12876-022-02106-2.

## Background

Screening colonoscopy for colorectal cancer (CRC) reduces CRC incidence and mortality due to the detection and removal of premalignant lesions [1]. Adequate bowel preparation is an essential pre-procedural clinical requirement that determines the diagnostic yield and therapeutic benefits of colonoscopy [2]. According to the European Society of Gastrointestinal Endoscopy (ESGE), the minimum standard for the rate of adequate bowel preparation is 90%, while the target standard is ≥ 95% [3]. However, in clinical practice these recommendations are usually unmet [4].

Generally, adequate cleansing is more difficult to attain in the right colon compared with other colon segments [5]. Moreover, the onco-protective effects of colonoscopy in the right colon are perceived to be relatively lower than those conferred by colonoscopy in the left colon [1, 6]. It has been hypothesised that inadequate cleansing can lead to missed lesions in the right colon, which is frequently found to have predominantly flat and non-pedunculated lesions [7–10]. Therefore, ensuring successful cleansing in the right colon is fundamental to maximising the protection against right-sided CRCs.

Clinical guidelines recommend both high- and low-volume polyethylene glycol (PEG)-based bowel preparations for routine bowel cleansing [11, 12]. Low-volume PEG-based cleansing agents, such as the 1L PEG-based bowel preparation NER1006, have been shown to be as effective as high-volume agents, but reduce the requirement for high-volume preparation intake and thus improve overall patient adherence to treatments [13].

In randomised clinical trials, NER1006 has shown superior rates of right colon high-quality cleansing versus 2L PEG plus ascorbate (2LPEG; the MORA study), and numerically higher rates of right colon high-quality cleansing versus oral sulphate solution (OSS; the NOCT study) [14, 15]. The improved cleansing efficacy of NER1006 in these trials is consistent with the results reported in a recent Italian prospective observational study, which enrolled 1289 patients to receive 4L, 2L or 1L PEG-based solutions for colon cleansing before undergoing colonoscopy [16]. The study reported superior cleansing in the right colon in patients who received NER1006 compared with other high-volume PEG-based bowel preparations.

Our aim in this post hoc analysis of data from the MORA and NOCT trials was to specifically examine whether NER1006 compared with 2LPEG and/or OSS can attain higher adequate-level and high-quality cleansing success rates in the right colon. Finally, we investigated the cleansing impact of NER1006 in the right colon in patients who are at increased risk of inadequate bowel preparation.

## Methods

### Patients

The MORA and NOCT trials included both male and female patients aged 18–85 years with varied demographic and clinical characteristics who required a screening, surveillance or diagnostic colonoscopy. This post hoc analysis mainly used data from the modified full analysis set 2 (mFAS2) of the two trials, which included all patients with full segmental Harefield Cleansing Scale (HCS) scorings by treatment-blinded central readers. The mFAS2 population only included patients who underwent colonoscopy with a colon-cleansing assessment, which better reflects a patient population undergoing colonoscopy by endoscopists in a real-world setting.

### Study design

This is a post hoc analysis of two parallel, randomised, multicentre, central reader treatment-blinded, Phase III clinical trials (MORA and NOCT) that assessed the bowel-cleansing efficacy, safety and tolerability of NER1006 relative to two other active bowel preparation comparators, preceding colonoscopy, in adults. The study methods involved in each of the clinical trials and the results obtained have been detailed previously [14, 15]. In MORA, patients were randomly assigned (1:1:1) to receive NER1006, administered either as a 2-day evening/morning split-dosing regimen (N2D) or as a same-day morning-only dosing regimen (N1D), or 2LPEG as a 2-day evening/morning split-dosing regimen. In NOCT, patients were randomly assigned (1:1) to receive NER1006 or OSS, each provided as a 2-day evening/morning split-dosing regimen. Product formulations are described in Additional file 1: Table S1. Both of the NER1006 regimens allowed a light breakfast and light lunch, and N1D allowed a light dinner. 2LPEG and OSS were administered as per their labels: 2LPEG permitted meals, including a light dinner (clear soup and/or plain yoghurt), while OSS allowed only breakfast on the day before colonoscopy. The first dose of each evening/morning split-dose regimen was started at 18:00 on the evening before the colonoscopy. After those dietary restrictions, patients could consume only clear fluids ad libitum until 2 h (NER1006 and OSS) or 1 h (2LPEG) before the start of the colonoscopy. Cleansing performance was initially evaluated by site endoscopists using the HCS, and then by central readers using video evaluation.

### Assessments

The main objective of this post hoc analysis was to assess the attainment of adequate-level cleansing success and also high-quality cleansing in the right colon, as assessed by central readers using the HCS, in patients with complete cleansing data.

We compared right colon adequate-level cleansing success rates for N2D (combined) and N1D versus 2LPEG or OSS; right colon adequate-level cleansing success rates for the combined N2D regimens versus the combined populations treated with 2LPEG or OSS (2LPEG/OSS).

To expand our understanding of high-quality cleansing success, we also assessed the following outcomes per treatment: comparative right colon high-quality cleansing success rate for N2D (combined) and N1D versus 2LPEG or OSS; right colon high-quality cleansing success rates for the combined N2D versus the 2LPEG/OSS population. Lastly, we studied the cleansing efficacy of N2D versus 2LPEG/OSS in the right colon of males with body mass index (BMI) ≥ 25 kg/m^2^_,_ as these patients are commonly known to be at increased risk for both inadequate cleansing and CRC [17]. The rates of right colon adequate and high-quality cleansing success with N2D versus 2LPEG/OSS were compared separately in overweight males and those without these risk factors for inadequate cleansing.

### Statistical analysis

For each trial, patient information and central reader-recorded HCS scores were extracted from the data collected to calculate the proportion of patients who had adequate cleansing success in the right colon and the proportion who had high-quality cleansing success in the right colon. Success rates are presented as percentages. The one-sided t-test was used to assess the superiority of NER1006 versus its comparators for both adequate and high-quality cleansing success rates. Finally, the one-sided t-test was used to determine the p-values for the combined analyses. Comparisons between overweight male patients and all other patients used two-sided t-tests, assuming equal variance. Right colon adequate and high-quality cleansing success rates with NER1006 versus comparators in overweight males and, separately, in all other patients, were compared using the one-sided t-test.

To assess the effect of variables that might influence the rates of adequate or high-quality right colon cleansing in the study population as a whole, we performed a multivariable logistical regression analysis with variables of: age, sex, BMI, study cohort, colonoscopy indication, time lapse between preparation completion and colonoscopy start, and NER1006 as the bowel preparation assigned. Two regression models were generated with adequate and high-quality right colon cleansing as the outcomes assessed.

A *p* < 0.05 was considered significant.

## Results

### Patient population

**Fig. 1:**
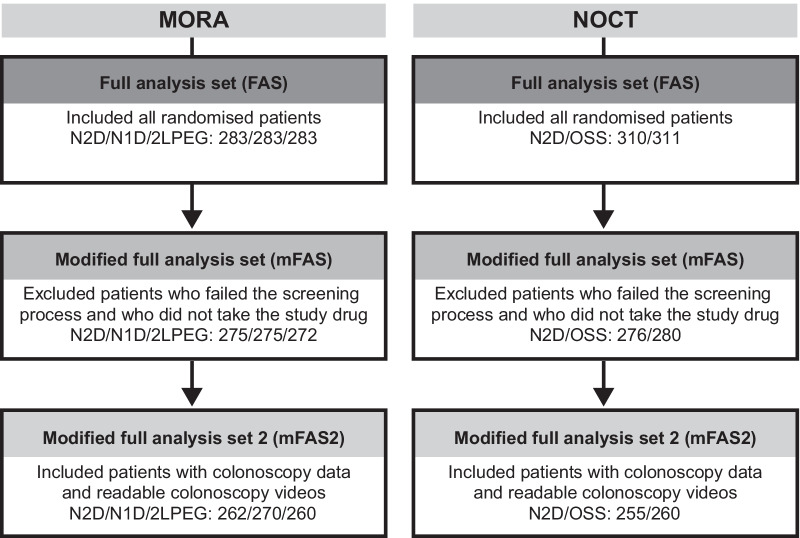
Patient disposition. 2LPEG, 2L polyethylene glycol plus ascorbate; N1D, NER1006 same-day morning-only dosing regimen; N2D, NER1006 2-day evening/morning split-dosing regimen; OSS, oral sulphate solution

Baseline characteristics of the mFAS2 population are summarised in Table 1. Baseline characteristics in mFAS2 were comparable across treatment arms, except for some imbalances observed in the sex and age distribution between the N2D and 2LPEG arms in MORA. Patient disposition is presented in Fig. 1. The mFAS2 population comprised 1307 patients, including 792 patients in MORA (N1D: 270; N2D: 262; 2LPEG: 260) and 515 patients in NOCT (N2D: 255; OSS: 260).

**Table 1:** Patient characteristics of the mFAS2 population (MORA and NOCT combined)

Characteristic	N2D (n = 517)	N1D (n = 270)	2LPEG (n = 260)	OSS (n = 260)
Sex, n (%)				
Female	280 (54.1)	145 (53.7)	123 (47.3)	115 (44.2)
Male	237 (45.8)	125 (46.3)	137 (52.7)	145 (55.8)
Age group, n (%)				
≤ 65 years	400 (77.4)	210 (77.8)	214 (82.3)	213 (81.9)
> 65 years	117 (22.6)	60 (22.2)	46 (17.7)	47 (18.1)
Race, n (%)				
White/Caucasian	473 (91.5)	267 (98.9)	257 (98.8)	215 (82.7)
Black	36 (7.0)	3 (1.1)	1 (0.4)	24 (9.2)
Asian	7 (1.4)	0	2 (0.8)	16 (6.2)
Other	6 (1.2)	0	0	5 (1.9)
BMI, n	514	268	256	260
Mean (kg/m^2^) (SD)	28.4 (5.3)	26.9 (4.3)	26.4 (4.1)	29.7 (6.2)
Patients with BMI > 25 kg/m^2^, n (%)	371 (72.2)	175 (65.3)	154 (60.2)	201 (77.3)
Colonoscopy indication, n (%)				
Screening	283 (54.7)	137 (50.7)	129 (49.6)	157 (60.4)
Surveillance	141 (27.3)	57 (21.1)	60 (23.1)	76 (29.2)
Diagnosis	93 (18.0)	76 (28.1)	71 (27.3)	27 (10.4)
Time lapse—preparation to colonoscopy, n	512	266	253	258
Mean (SD) time (hours) between the last dose of bowel preparation and colonoscopy	5.6 (1.9)	5.4 (1.7)	5.4 (2.1)	5.3 (2.1)

2LPEG, 2L polyethylene glycol plus ascorbate; BMI, body mass index; mFAS2, modified full analysis set 2; N1D, NER1006 same-day morning-only dosing regimen; N2D, NER1006 2-day evening/morning split-dosing regimen; OSS, oral sulphate solution; SD, standard deviation

### Adequate-level cleansing success

#### Comparative right colon adequate-level cleansing success

**Fig. 2:**
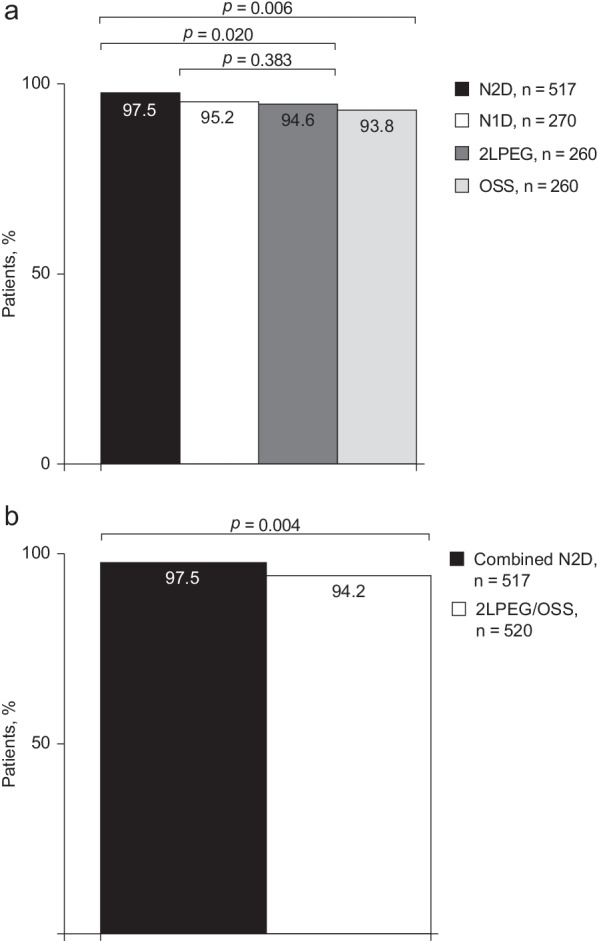
Right colon adequate-level cleansing rates **a** overall; and **b** with combined N2D regimens and 2LPEG/OSS. 2LPEG, 2L polyethylene glycol plus ascorbate; N1D, NER1006 same-day morning-only dosing regimen; N2D, NER1006 2-day evening/morning split-dosing regimen; OSS, oral sulphate solution

Among patients who had their bowel preparations administered as an evening/morning split-dose regimen, pooled N2D data showed significantly improved adequate-level cleansing in the right colon compared with 2LPEG (97.5% [504/517] versus 94.6% [246/260]; *p* = 0.020) and OSS (97.5% [504/517] versus 93.8% [244/260]; *p* = 0.006; Fig. 2a). In the MORA study, rates of adequate right colon cleansing did not significantly differ between N1D and 2LPEG (95.2% [257/270] versus 94.6% [246/260]; *p* = 0.383).

#### Combined analysis

Significantly superior rates of adequate-level cleansing success were shown in combined data from the N2D regimens in MORA and NOCT versus combined 2LPEG/OSS (97.5% [504/517] vs 94.2% [490/520], *p* = 0.004) (Fig. 2b).

### High-quality cleansing success

#### Comparative right colon high-quality cleansing success

**Fig. 3:**
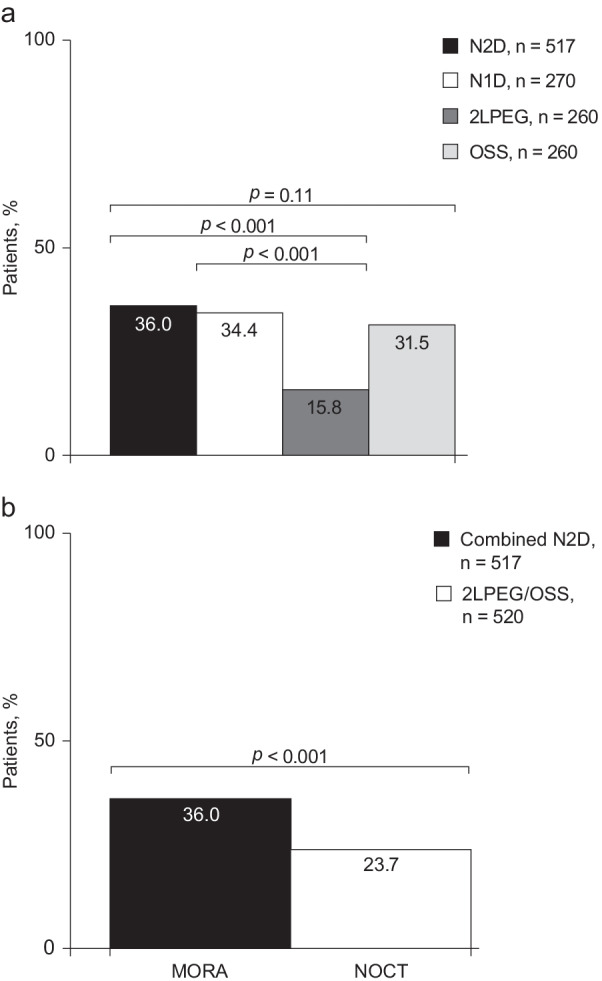
Right colon high-quality cleansing rates **a** overall; and **b** with combined N2D regimens and 2LPEG/OSS. 2LPEG: 2L polyethylene glycol plus ascorbate; N1D, NER1006 same-day morning-only dosing regimen; N2D, NER1006 2-day evening/morning split-dosing regimen; OSS, oral sulphate solution

Analysis of the evening/morning split-dose preparations showed that patients receiving an N2D regimen had a significantly higher rate of high-quality cleansing than patients receiving 2LPEG (36.0% [186/517] versus 15.8% [41/260]; *p* < 0.001), and a numerically higher rate than patients receiving OSS (36.0% [186/517] versus 31.5% [82/260]; *p* = 0.11). In MORA, rates of high-quality cleansing were significantly improved with N1D compared with 2LPEG (34.4% [93/270] versus 15.8% [41/260]; *p* < 0.001) (Fig. 3a).

#### Combined analysis

Combined data from MORA and NOCT showed significantly higher rates of right colon high-quality cleansing success with N2D versus 2LPEG/OSS (36.0% [186/517] vs 23.7% [123/520]; *p* < 0.001) (Fig. 3b).

### Right colon-cleansing success in overweight males

#### Adequate-level cleansing success in N2D and 2LPEG/OSS

**Fig. 4:**
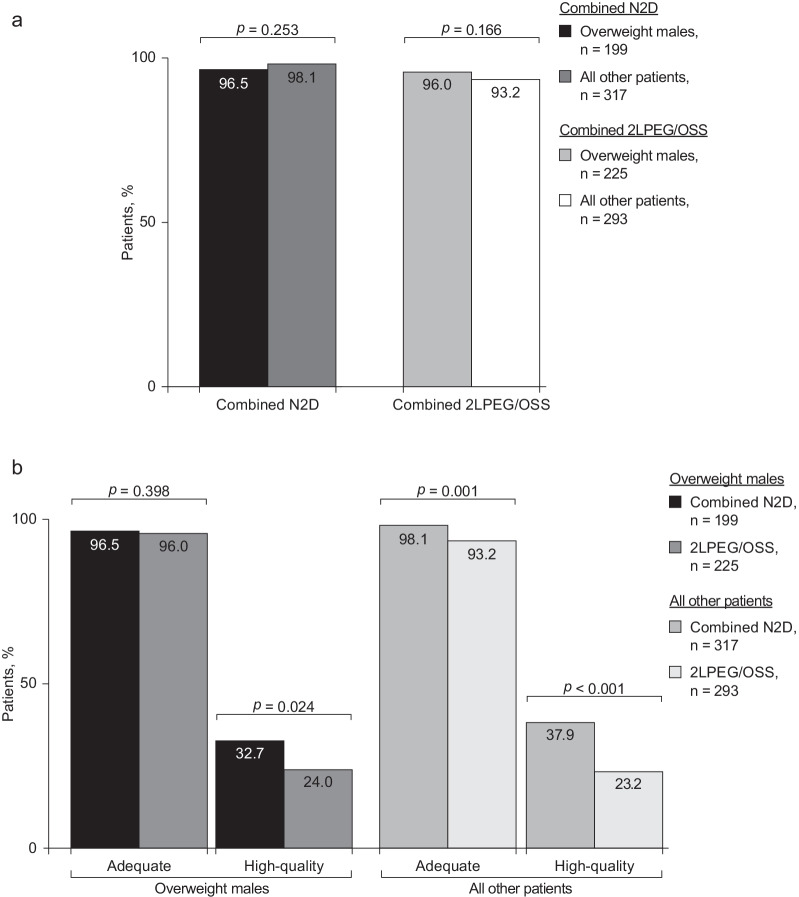
Right colon cleansing in overweight males by **a** treatment regimen (adequate cleansing); **b** patient category. 2LPEG, 2L polyethylene glycol plus ascorbate; N2D, NER1006 2-day evening/morning split-dosing regimen; OSS, oral sulphate solution

Treatment with NER1006 enabled overweight male colonoscopy patients to attain an adequate-level cleansing success rate in the right colon that was comparable with that of the rest of the patient group (96.5% [192/199] versus 98.1% [311/317]; *p* = 0.253; Fig. 4a). With 2LPEG/OSS, the adequate-level cleansing success rate in the right colon was comparable between overweight male patients and all other patients, with no statistically significant difference observed between the groups (96.0% [216/225] versus 93.2% [273/293]; *p* = 0.166; Fig. 4a).

#### Adequate and high-quality cleansing success in overweight males

In the combined N2D group, a numerically higher proportion of overweight male patients attained adequate-level cleansing success in the right colon versus the 2LPEG/OSS group (96.5% [192/199] versus 96.0% [216/225]; *p* = 0.398; Fig. 4b).

The proportion of overweight male patients with high-quality cleansing success in the right colon was significantly higher in the N2D group compared with the 2LPEG/OSS group (32.7% [65/199] versus 24.0% [54/225]; *p* = 0.024; Fig. 4b).

#### Adequate and high-quality cleansing success in all other patients

In all other patients, the rate of right colon adequate cleansing was superior with N2D versus 2LPEG/OSS (98.1% [311/317] versus 93.2% [273/293]; *p* = 0.001; Fig. 4b). A significantly higher proportion of patients attained high-quality cleansing in the right colon in the N2D group versus the 2LPEG/OSS group (37.9% [120/317] versus 23.2% [68/293]; *p* < 0.001; Fig. 4b).

### Multivariable logistic regression on right colon cleansing

#### Adequate right colon cleansing

In the multivariable logistical regression analysis for factors influencing an outcome of adequate right colon cleansing, there was a significant positive association between adequate cleansing and assignment of NER1006 as the bowel preparation (odds ratio 1.87; 95% confidence interval [CI] 1.06–3.33; *p* = 0.031). There was a negative association between the time lapse from preparation to colonoscopy and adequate cleansing (OR 0.88; 95% CI 0.79–1.00; *p* = 0.034).

#### High-quality right colon cleansing

In the regression analysis with high-quality right colon cleansing as the outcome, a positive association with NER1006 use was again observed (OR 1.98; 95% CI 1.53–2.58; *p* < 0.001). Longer time lapses between preparation and colonoscopy were associated with lower high-quality cleansing rates (OR 0.91; 95% CI 0.85–0.97; *p* = 0.006).

## Discussion

This post hoc analysis assessed whether the low-volume PEG-based bowel preparation 1L NER1006 could improve right colon adequate-level and high-quality cleansing success compared with two medium-volume alternatives among patients with complete segmental cleansing data from two randomised controlled trials.

Adequate-level cleansing success is a vital quality metric in colonoscopy [18], and poor bowel preparation can result in missed diagnoses and thereby delay initiation of treatment [19]. Inadequately cleansed patients should, per guideline recommendations, undergo early repeat colonoscopy. Reducing the frequency of early repeat colonoscopies may improve cost effectiveness and also improve patient willingness to undergo the procedure in the future [20–24]. In addition, this inefficiency can lead to delays in attending to other patients on the waiting list.

The right colon is particularly difficult to cleanse to an optimum level for colonoscopy as, following stool clearance from the colon, mucus and chyme that are secreted from the small intestine tend to stick to the caecum and right colon [10]. This increases the risk of missed lesions in the right colon, particularly flat, non-pedunculated lesions and sessile serrated adenomas, which may have a higher malignant potential [7–10, 25]. In our analysis, pooled data from the evening/morning split-dose NER1006 regimens demonstrated a statistically significant higher rate of right colon adequate-level cleansing versus 2LPEG and OSS individually. Compared with 2LPEG, the low volume of NER1006 is achieved by increasing the ascorbate components of the bowel preparation and including them in the second administered dose [26]. This increases the osmotic activity of the preparation relative to 2LPEG and also enables delivery in a total preparation volume of 1L [14]. This enhanced osmotic activity may contribute to improved cleansing of the right colon, as seen in the original studies and detailed in these post hoc analyses [14–16, 27, 29–31].

High-quality cleansing, as assessed by the HCS, is associated with numerically improved adenoma detection in the right colon and significantly improved adenoma detection in the overall colon [27]. In its Phase III clinical development programme, NER1006 demonstrated numerically improved high-quality cleansing success rates in the right colon versus all of its comparators and a statistically significant improvement over 2LPEG [14, 15]. In a prospective observational study, a higher proportion of hospitalised patients attained high-quality cleansing in the right colon with NER1006, which was statistically significant, compared with 4LPEG [28]. In the current post hoc analysis, the superior right colon high-quality cleansing success rates obtained with NER1006 in the mFAS2 population are therefore consistent with, and an important clarification of, these previous results.

The superior high-quality cleansing success rate with N2D, when assessed strictly by central readers, is consistent with the previously reported superior overall high-quality cleansing success of N2D versus 2LPEG or OSS as assessed by site endoscopists [29]. Furthermore, the adequate-level and high-quality cleansing rates attained in the right colon in the combined populations of N2D across both the MORA and NOCT trials were superior to those seen in the combined population of patients treated with 2LPEG or OSS (2LPEG/OSS).

Several risk factors contributing to inadequate bowel cleansing have been identified in the literature, and overweight men are regarded as being at high risk [30]. This specific category of patients is also considered to be at increased risk of CRC, with a high prevalence of colorectal adenomas and polyps in this population [31]. In line with previous studies on cleansing efficacy in high-risk patients, in this study, NER1006 effectively delivered comparable levels of right colon adequate cleansing in both overweight males and all other patients [30]. Similar findings were observed in the 2LPEG/OSS group; however, a numerically higher rate was reported in patients treated with NER1006. The superior overall high-quality cleansing efficacy of NER1006 versus 2LPEG/OSS in overweight men and, separately, in obese male patients older than 60 years, has been reported previously [30, 31]. In the current study, NER1006 maintained its high-quality cleansing superiority versus 2LPEG/OSS in the right colon of hard-to-cleanse patients.

This study has several strengths. It is based on two randomised Phase III clinical trials conducted across multiple centres in the USA and Europe to evaluate the cleansing efficacy of the first 1L PEG-based product, NER1006, versus two mid-volume bowel preparations. Importantly, the MORA and NOCT trials were designed with near-identical study protocols, with cleansing assessed by both treatment-blinded endoscopists and central readers using a validated colon-cleansing scale, the HCS. These clinical trials were also the first to be optimised for assessing high-quality cleansing of the right colon as a primary endpoint. The mFAS2, the population set used in this analysis, closely resembles the patient population undergoing colonoscopy in real-world clinics.

Our study has limitations. The major source of limitation is the post hoc analysis. The noticeably lower high-quality cleansing success rates observed in this analysis compared with the adequate-level cleansing rates attained in the right colon are due to the criteria strictly applied by central readers for defining high-quality cleansing on the HCS; the perceived cleansing quality by site endoscopists tends to be higher [13, 29].

In conclusion, in patients who underwent colonoscopy with full segmental scorings using the HCS by treatment-blinded central readers, N2D demonstrated improved adequate and high-quality cleansing of the right colon compared with 2LPEG and OSS. NER1006 successfully delivered comparable high levels of right colon cleansing in overweight males and all other patients. These cleansing benefits of NER1006 are promising, and will hopefully help healthcare practitioners to further enhance the diagnostic and therapeutic efficacy of colonoscopy in the right colon.

## Supplementary Information

**Additional file 1.** Product formulations of NER1006, OSS and 2LPEG.

## Data Availability

All data generated or analysed during this study are available from the corresponding author on reasonable request.

## Abbreviations

2LPEG: 2L PEG plus ascorbate
BMI: Body mass index
CI: Confidence interval
CRC: Colorectal cancer
ESGE: European Society of Gastrointestinal Endoscopy
HCS: Harefield Cleansing Scale
mFAS2: Modified full analysis set 2
OR: Odds ratio
N1D: NER1006 same-day morning-only dosing regimen
N2D: NER1006 2-day evening/morning split-dosing regimen
OSS: Oral sulphate solution
PEG: Polyethylene glycol
