# Supplementary material for: 1L NER1006 can improve rates of adequate and high-quality bowel cleansing in the right colon: a post hoc analysis of two randomised clinical trials
Source: BMC Gastroenterol. 2022 Jan 25;22:35. doi: 10.1186/s12876-022-02106-2 (PMC8787873; doi:10.1186/s12876-022-02106-2)
Supplement: Supplementary file 1 — Additional file 1. Product formulations of NER1006, OSS and 2LPEG. [file 12876_2022_2106_MOESM1_ESM.docx]

**Supplementary information**

**Supplementary Table 1** Product formulations of NER1006, OSS and 2LPEG

|  | NER1006^a^ | | 2LPEG | | OSS | |
| --- | --- | --- | --- | --- | --- | --- |
|  | Dose 1 | Dose 2 | Dose 1 | Dose 2 | Dose 1 | Dose 2 |
| Osmotically active ingredients, g |  | | | | | |
| PEG3350 | 100 | 40 | 100 | 100 | – | – |
| Sodium sulphate | 9 | – | 7.5 | 7.5 | 17.5 | 17.5 |
| Potassium sulphate | – | – | – | – | 3.1 | 3.1 |
| Magnesium sulphate | – | – | – | – | 1.6 | 1.6 |
| Sodium ascorbate | – | 48.1 | 5.9 | 5.9 | – | – |
| Ascorbic acid | – | 7.5 | 4.7 | 4.7 | – | – |
| Bowel preparation volume, mL | 500 | 500 | 1000 | 1000 | 473 | 473 |
| Additional fluid volume, mL | 500 | 500 | 500 | 500 | 946 | 946 |
| Total volume, mL | 1000 | 1000 | 1500 | 1500 | 1419 | 1419 |
| Total preparation volume, mL | 2000 | | 3000 | | 2838 | |

^a^ Patients are permitted to drink additional clear fluids ad libitum [14, 15].

2LPEG: 2L polyethylene glycol plus ascorbate; OSS: oral sulphate solution.
